# Modulation of Immune Response by Organophosphorus Pesticides: Fishes as a Potential Model in Immunotoxicology

**DOI:** 10.1155/2015/213836

**Published:** 2015-04-20

**Authors:** K. J. G. Díaz-Resendiz, G. A. Toledo-Ibarra, M. I. Girón-Pérez

**Affiliations:** Universidad Autónoma de Nayarit, Secretaría de Investigación y Posgrado, Laboratorio de Inmunotoxicología, Ciudad de la Cultura s/n, 63000 Tepic, NAY, Mexico

## Abstract

Immune response is modulated by different substances that are present in the environment. Nevertheless, some of these may cause an immunotoxic effect. In this paper, the effect of organophosphorus pesticides (frequent substances spilled in aquatic ecosystems) on the immune system of fishes and in immunotoxicology is reviewed. Furthermore, some cellular and molecular mechanisms that might be involved in immunoregulation mechanisms of organophosphorus pesticides are discussed.

## 1. Introduction

Organophosphorus pesticides (OPs) are a group of insecticides derived from the phosphoric or phosphorothioic acid; its use has increased in the recent years for the improvement of agriculture production, in the industry and prevention of human health through control and/or eradication of unwanted insects, plants, animals, and disease vectors [1]. According to information published by the National Pesticide Information Center (NPIC), the most used OPs are chlorpyrifos, malathion, acephate, naled, dicrotophos, phosmet, phorate, diazinon, dimethoate, and azinphos-methyl. At a worldwide level, an average of 37 million pounds of these active substances is sold [2].

Even though OPs have limited persistence in the environment, they are highly toxic for humans and are responsible for most of accidental intoxications [3]. The World Health Organization (WHO) estimated that, every year, almost 3 million people suffer acute intoxication due to OPs [4]; hence its use is considered a worldwide public health problem [5]. OPs cause two main toxic effects. The first one is acute toxicity, initiated by the inhibition of the acetylcholinesterase enzyme (AChE) with the subsequent accumulation of acetylcholine (ACh) in the nervous termination, provoking an overstimulation of muscarinic acetylcholine (mAChR) and nicotinic acetylcholine (nAChR) receptors. The inhibition mechanism of AChE is conducted through phosphorylation of the hydroxyl group in the serine of the active site of the enzyme; once phosphorylated it is extremely stable, which avoids its physiological action on the ACh that consists in the degradation of this neurotransmitter to allow reuptake of acetate and choline in the nervous terminal [3, 6, 7]. The second effect is chronic and is denominated organophosphate-induced delayed polyneuropathy (OPIDP), which is characterized by ataxia and paralysis, signs that appear 2-3 weeks after exposure to OPs [1, 8].

### 1.1. Effects of OPs in Aquatic Ecosystems and Fishes

After its application on agricultural crops, residual OPs enter water bodies as result of spray drift, soil leaching, and running off soils dedicated to agriculture, provoking adverse effects on the target species but also on a wide range of nontarget organisms, especially those that inhabit aquatic ecosystems such as invertebrates, birds, and fishes [9, 10].

Among the nontarget species exposed to OPs, it is important to mention fishes, since these organisms are transcendental due to their status as top consumer species in the food chain, besides of playing an important role in the maintenance of the balance of aquatic ecosystems. From an evolutionary point of view, fishes are important organisms because they appeared over 560 million years ago; they are a group of vertebrates phylogenetically antique; there are over 25,000 species; therefore their great diversity stands out in comparison to other vertebrates [11, 12]. Among other species, some have stood out for their ecological or economic importance, while others have been used as study models in diverse areas of scientific research [13].

## 2. Immune System of Fishes

Fishes are the first group of organisms that present an innate and adaptive immunity system; therefore the study of these organisms is of great relevance due to the information it gives about evolution of the immune system in vertebrates [14].

The innate immune system is of paramount importance in fishes [15–17]; among the components of humoral innate immunity that are mainly characterized in fishes are antibacterial peptides, lysozymes, lectins, acute-phase proteins, and molecules of the complement system, while innate immunity cells mostly characterized are macrophages, neutrophils, and eosinophils [18–20].

On the other hand, adaptive immunity mechanisms in fishes play a vital role in the protection against recurrent infections, response that is mediated by T- and B-lymphocytes and antibodies. Fishes are the first vertebrates where clonal selection and genetic rearrangement in receptors of lymphocytes are present. Likewise, leucocytes with T cell activity have been reported, similar to the cooperative and cytotoxic T cells of mammals (CD4^+^-like, CD8^+^-like). Apart from that, based on the profile of cytokines, there have been reports of T cells subpopulations similar to the ones reported in mammals [21]. In contrast, B cells in fishes have been characterized through the expression of antigen receptor (BCR). In fishes, IgM is the main soluble antibody, which is tetrameric; on the other hand, IgD, just like in mammals, is expressed in the surface of B cells. In addition, other isotypes have been identified, such as lgT and lgZ, which are mainly found in mucosa, such as in intestine, in skin, and in gills [21–24].

## 3. OPs Immunotoxicity in Fishes

In recent years, an immunotoxic effect of OPs has been reported in diverse organisms, including fishes. Immune system is the first defense line against pathogenic organisms; however, it is a very sensitive system to be altered by stressing factors present in the environment (biotic and abiotic) [1, 10]; thus it is vulnerable to any xenobiotic such as OPs, which can cause structural or functional alterations in humoral or cell mechanisms (nonspecific or adaptative) of the immune response (Table 1), which entails, among others, an increase in the susceptibility to infections [6].

### 3.1. OPs and Humoral Response

In fishes, molecules that are responsible of the innate and adaptative humoral response can be altered by OPs, like chlorpyrifos, diazinon, and phosalone, among others [10, 25–30].

Thus, lysozyme is an important molecule defense of the innate immune system of fishes that is frequently altered by OPs. A study showed that the lysozyme activity increased significantly in liver and spleen of beluga (*Huso huso*) exposed acutely to diazinon (1.5 mg/L). However, at subacute and subchronic exposure of this pesticide, lysozyme activity decreased in plasma, liver, kidney, and spleen [31]. On the other hand, it has been reported that the acute exposure to diazinon (2.0 and 4.0 mg/L) in grass carp (*Ctenopharyngodon idella*) induced a significant increase in the lysozyme activity present in kidney and spleen of this fish. Nevertheless, in plasma of these organisms, enzyme activity diminished significantly [25]. Recent studies have reported a decrease in the lysozyme activity in plasma of rainbow trout (*Oncorhynchus mykiss*) and common carp (*Cyprinus carpio* L.) exposed to diazinon (0.1 and 0.2 mg/L) and phosalone (0.15, 0.30, and 0.60 mg/L), respectively [28, 30]. Also, it has been reported that chlorpyrifos provoke a diminishment in the enzyme activity present in plasma and spleen of common carp (*C. carpio*) exposed acutely to 75 *μ*g/L of pesticide [10]. Recently, it was reported that exposure of Nile tilapia (*Oreochromis niloticus*) to chlorpyrifos (0.102 and 0.255 mg/L) provoked an increase in the activity of this enzyme in the plasma of these organisms; however, at a lower concentration (0.051 mg/mL) the pesticide did not cause any effect on the activity of this enzyme [29].

Another important molecule of the innate immune system of fishes is the protein C3 of the complement, which is also altered by the exposition to OPs. A deregulation at concentration and mRNA expression of this molecule has been reported in anterior kidney, spleen, and plasma of common carp (*C. carpio* L.) exposed acutely to chlorpyrifos [10].

Reactive C protein (RCP) is another molecule of the innate immune system of fishes affected by exposure to this type of pesticides. In this context, it has been reported that acute exposure to metrifonate (0.4 ppm) in rainbow trout (*O. mykiss*) provoked a significant increase of this protein in the plasma of organisms exposed during 3 days to the pesticide; however, at 10 and 18 days after exposure, protein activity diminished significantly [32].

Other proteins that are also altered by the exposure to OPs are the globulins. Some studies have reported that, in plasma of rainbow trout (*O. mykiss*), concentration of these proteins diminishes significantly when organisms are exposed acutely and subacutely to diazinon (0.1 and 0.2 mg/L) [27, 28]. Likewise, a diminishment in the concentration of globulins in plasma of common carp (*C. carpio* L.) exposed acutely to phosalone has been shown [30]. On the other hand, it has been reported that immunoglobulins are also affected by OPs; in this sense, there are studies that show that these pesticides alter the concentration of IgM, which is the most important gamma-globulin in fishes [33, 34]. In this context, it has been published that chlorpyrifos (0.051 mg/mL) diminish concentration of IgM in plasma of Nile tilapia (*O. niloticus*) [29]. Furthermore, it has also been reported that the exposure to chlorpyrifos (75 *μ*g/L) during 24 h provoked a significant decrease of IgM in plasma of common carp, apart from a diminishment of IgM present in spleen of fishes exposed acutely to 15 and 75 *μ*g/L of the pesticide [10]. In addition, a significant increase has been reported in the concentration of IgM in plasma of Nile tilapia exposed acutely to diazinon (1.96 mg/L). Nevertheless, exposure to lower concentrations of this pesticide (0.78 and 0.39 mg/L) did not alter concentration of IgM in plasma of these organisms [26, 35].

Regarding the effect of OPs on the cytokines, it has been reported that the exposure to chlorpyrifos during 24 h (1.16, 11.6 and 116 *μ*g/L) induces an increase in the expression of mRNA of IL-1*β*, IL-1R, and IFN-*γ* in spleen of carp (*C. carpio*) [36].

### 3.2. OPs and Cellular Immune Response

The innate and adaptative cellular response of fishes can be deregulated by the exposure to diverse OPs. Studies show that exposure of rainbow trout and common carp to diazinon provokes a diminishment in the white blood cell (WBC) in these species. The differential account of these cells showed a diminishment in the percentage of lymphocytes, monocytes, and basophils; however, the percentage of neutrophils and eosinophils increased significantly after exposure to the pesticide [28, 37]. A decrease in WBC in other species such as Nile tilapia (*O. niloticus*) exposed to malathion (0.23 and 0.46 mg/L) and carp (*C. carpio*) exposed to phosalone (0.15, 0.30, and 0.60 mg/L) has also been reported. In common carp, lymphocytes diminished significantly at the three evaluated concentrations, even though the percentage of monocytes and neutrophils increased [1, 30]. In contrast to the results in the before mentioned studies, Ural [38] reported an increase in the WBC of common carp exposed to chlorpyrifos (0.04 and 0.08 mg/L). In this sense, Hedayati and Tarkhani [39] reported that, in iridescent shark (*Pangasius hypophthalmus*) exposed to diazinon (0.5 and 1 ppm), a significant increase in the total number of WBC, particularly in neutrophils, was shown, while the number of lymphocytes did not show any change due to the exposure to this pesticide. However, no eosinophils and monocytes were detected in the blood samples of the analyzed fishes [38, 39].

On the other hand, it was also reported that the OPs not only induce alteration in the number of cells, but also in the morphology and functionality of them. Hence, it was reported that diazinon (15, 30, 45, 60, and 75 *μ*g/L) provoked changes in the size of macrophages of kidney and spleen of the fish* Lepomis macrochirus* [40]. In addition, it has been reported that the phagocytic activity of cells is also altered by the exposure to OPs. Girón-Pérez et al. showed that the phagocytic index of mononuclear cells of Nile tilapia decreased by exposure* in vivo* to diazinon; however, an increase in the respiratory burst of these cells was observed [26, 35]. Regarding the effect of OPs on the proliferative capacity of lymphocytes, it has been reported that chlorpyrifos at concentrations 0.051, 0.102, and 0.255 mg/L during 96 h did not affect the proliferative capacity of lymphocytes in Nile tilapia [29]. However, the lymphoproliferation of splenocytes of this fish diminishes significantly after exposure* in vivo* to diazinon (7.83, 3.91, and 1.95 mg/L) during 96 h [35]. Nevertheless, the exposure* in vivo* of lymphocytes to diazinon and diazoxon (main metabolite of diazinon) did not affect the proliferative capacity of these cells [41].

## 4. Mechanisms of Immunotoxicity of OPs

**Figure 1:**
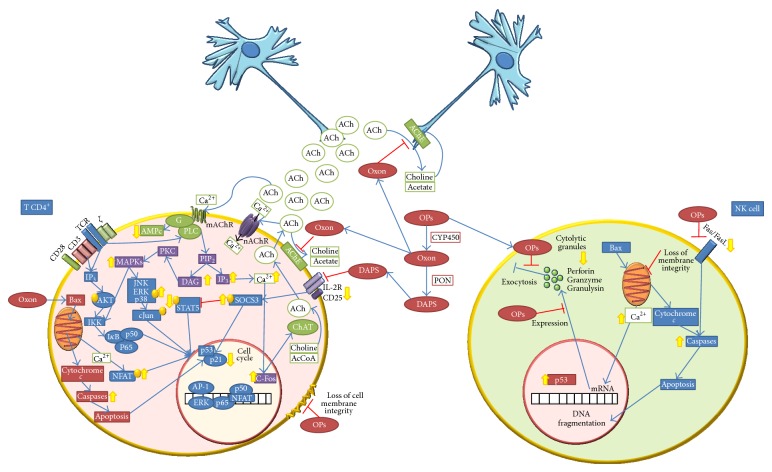
Mechanisms of immunotoxicity of OPs. The potential immunotoxicity mechanisms could involve effect directly on immune cells or through neuroimmune communication disturbance. AcCoA: acetyl-coenzyme A; ACh: acetylcholine; AChE: acetylcholinesterase; ChAT: acetylcholine transferase; CYP450: Cytochrome P450; DAPS: dialkylphosphates; mAChR: muscarinic acetylcholine receptor; nAChR: nicotinic acetylcholine receptor; OPs: organophosphorus pesticides; oxon: oxidized metabolite of OPs; PON: paraoxonase.

The effects mentioned above show that OPs alter the function of certain elements of the immune system, even though the mechanisms of immunotoxicity of the OPs are not clear. Such mechanism of OPs is not direct but it works through indirect mechanisms, topics that will be discussed in this section, based on evidence shown in different animal models (Figure 1).

### 4.1. OPs and Cholinergic Regulation

As previously mentioned, OPs are substances that have as target molecule the enzyme AChE, blocking its activity through the irreversible bound to the active site, which provokes an increase in the levels of the neurotransmitter ACh in the nervous system. In this context, in mammals, the influence of the nervous system on the regulation of the immune system has been demonstrated years ago [42]; thus the increase in the concentration of neurotransmitters, in this case neuronal ACh, can deregulate the immune function. Apart from that, there is clear evidence that lymphocytes of mammals express mAChR and nAChR in their membrane and possess all necessary enzymes to produce ACh and autodegrade it through the AChE enzyme; hence they possess a self-cholinergic system, denominated extraneuronal or nonneuronal cholinergic system [43, 44].

In this way, the existence of an extraneuronal cholinergic system in lymphocytes makes them susceptible to perturbation by OPs. It has been suggested that OPs can modulate lymphocytes through cholinergic receptors, evoking an immediate intracellular signalization of diverse molecules, among them c-Fos, modulating therefore the levels of second messengers. Activation of cholinergic receptors can act upstream in the transduction of signals, causing the interruption of cellular homeostasis, decaying into apoptosis [45]. Data obtained in our laboratory have proven that exposure* in vitro* to diazinon and diazoxon does not alter the lymphoproliferative capacity in fishes; nevertheless these substances induce an increase in the concentration of ACh, which significantly diminishes lymphoproliferation [41].

### 4.2. OPs and Cytotoxic Activity

Besides inhibiting the enzyme AChE, OPs are capable of inhibiting serine hydrolases enzymes, such as molecules of the complement and thrombin system, which will influence directly the functionality of the immune system. In addition, the damage in the lymphoid tissue is the result of the phosphorylation, oxidative damage, and/or altered neuronal function, induced by OPs [46]. In this sense, in aquatic and human models, it has been reported that OPs diminish NK cell, LAK cell, and cytotoxic activities [47–50]. Even though there are very few studies on the mechanisms of induced inhibition by OPs in this type of cells, it has been proposed that this effect might be mediated by the inhibition of serine proteases (granzymes, perforins, and granulysins), molecules that are usually released by exocytosis [47, 51–53]. In addition, OPs have been reported not only to inhibit activity and release granules, but also to inhibit the expression of genes related with these molecules [49, 50]. On the other hand, the effect of OPs in NK cells through FasL/Fas pathway has been researched, and it has been reported that dichlorvos (DDVP) induces decrease on the expression of Fas in cells YAC-1 and the expression of FasL in LAK cells. This suggests that OPs reduce cytolytic capacity and proapoptotic signals through two mechanisms: (1) diminishment in exocytosis of granules and (2) FasL/Fas pathway [46].

### 4.3. OPs and Transduction of Signals

Alterations of the components and immune functions have also been related to the sequence and intensity of phosphorylation and dephosphorylating of protein kinases, essential process to modulate the immune response. A key molecule in this process is the protein suppressor of cytokine signaling 3 (SOCS3), which regulates protein STAT. SOCS3 mediates inhibition of phosphorylation of STAT5, which has been related with the diminishment of cellular proliferation [46]. In this context, it has been reported that dialkylphosphates (DAPs), metabolites produced during biotransformation of OPs, interact with leucocytes altering cellular signalization. There is evidence that diethyldithiophosphate (DEDTP) and diethylthiophosphate (DETP) interact and produce effects on the immune system, reducing the expression of CD25 and CD4 and secretion of IL-2, altering signalization of IL-2R, by modifying the phosphorylation status of STAT5 proteins. Apart from that, it has been reported that DEDTP increases phosphorylation of SOCS3 and dephosphorylation of STAT5 and also induces phosphorylation of ERK, JNK, and p38, depending events of PKC, PLC-*γ*, and AMP-responsive element-binding protein, which results in the nuclear translocation of NFAT, AP1, and ERK [46, 54, 55]. Lima and Vega [56] reported that DEDTP induces the arrest of the cellular cycle, mediated by SOCS3, initiating a feedback mechanism associated with p21 and p53 [56, 57]. It has also been reported that OPs (chlorpyrifos, sarin, and soman) can activate the PLC-*γ* and after that the transduction via of signals MAPK through PKC, as a consequence of accumulation of IP3 and DAG [58].

### 4.4. OPs and Apoptosis

Some studies have suggested the implication of OPs in apoptotic processes. It is known that the initiation of apoptosis is regulated by external and internal signals, such as the activation of dead receptors, damage to DNA, and perturbation of the mitochondrial membrane. These mechanisms carry the caspases activation and subsequently the destruction of the cell in a programmed way [59]. Thus, it has been reported that some OPs (monocrotophos, profenofos, chlorpyrifos, and acephate) induce apoptosis and necrosis in cultured human lymphocytes of peripheral blood [60]. Nakadai et al. reported that chlorpyrifos induce apoptosis in the cellular line U937 of human monocytes, besides inducing an increase of caspase 3 [61]. On the other hand, it has been shown that parathion and paraoxon (parathion metabolite) induce apoptosis in the cellular line of lymphocytic leukemia T (EL4) through activation of caspase-3. Likewise, exposure* in vitro* and* in vivo* to paraoxon provoked cytochrome C translocation from the mitochondria to cytosol, activating proapoptotic molecules such as Bax [59]. It has been shown that exposure of cell line ZC-7901 of grass carp fish (*Ctenopharyngodon idellus*) to malathion (23.75 mg/L) during 2 h induces a decrease in the mitochondrial membrane potential (ΔΨm), besides increasing the intracellular calcium flux [62].

## 5. Conclusion

Fishes are the first vertebrates with innate and adaptative immune mechanisms, similar to mammals. Thus, fishes can be used as a model in biomedical research, allowing data in the immunotoxicology field in evolutionary terms. Besides, due to fishes being the most abundant vertebrate in the planet, a lot of them with commercial importance, data generated could have economic and ecological importance.

There are evidences that the immune response can be altered by OPs exposure. Although, the immunotoxicity mechanisms are not completely clarified, evidence suggests that OPs can target several molecules related to the immune system and execute the immunotoxic effect through the alteration of the neuroimmune communication, particularly the cholinergic neuronal and immune system. Nevertheless, further research is needed in order to understand the mechanisms of immunoregulation of this type of pesticides widely used in household and agricultural activities.

## Figures and Tables

**Table 1:** Toxic effects of OPs in humoral and cellular immune parameters in fishes.

Parameters	OP	Effects	Tissue/cell line	Exposure time	Concentration	Species	References
Humoral immune response							

Lysozyme activity	Diazinon	↑ ↓ ↓	Spleen	1, 7 d 21, 28 d 35, 42, 49, 56, 63 d	1.5 mg/L	Great sturgeon or beluga (*Huso huso*)	[31]
		↓	Plasma	35, 42, 56, 63 d			
		↑	Kidney	63 d			
		↑ ↓ ↑	Spleen Plasma Kidney	7 d 1 d 7 d	2.0, 4.0 mg/L	Grass carp (*Ctenopharyngodon idella*)	[25]
		↓	Plasma	15, 30 d	0.1, 0.2 mg/L	Rainbow trout (*Oncorhynchus mykiss*)	[28]
	Chlorpyrifos	↑ ↓	Spleen	3 d 7 d	15 *µ*g/L 75 *µ*g/L	Common carp (*Cyprinus carpio*)	[10]
		↑ ↓	Plasma	3 d 3, 5, 7 d	15 *µ*g/L 75 *µ*g/L		
		↑ ↑	Kidney	3 d 5 d	15 *µ*g/L 75 *µ*g/L		
		↑	Plasma	4 d	0.102, 0.255 mg/mL	Nile tilapia (*Oreochromis niloticus*)	[29]
	Phosalone	↓	Plasma	7, 14 d	0.15, 0.30, 0.60 mg/L	Common carp (*Cyprinus carpio*)	[30]

C-reactive protein	Metrifonate	↑ ↓	Plasma	3 d 10, 18 d	0.4 ppm	Rainbow trout (*Oncorhynchus mykiss*)	[32]

Globulin	Diazinon	↓	Plasma	7, 14, 28 d	0.1, 0.2 mg/L	Rainbow trout (*Oncorhynchus mykiss*)	[27]
		↓		7, 15, 30 d			[28]
	Phosalone	↓	Plasma	14 d	0.15, 0.30, 0.60 mg/L	Common carp (*Cyprinus carpio*)	[30]

IgM	Diazinon	→	Plasma	4 d	3.915, 7.830 ppm	Nile tilapia (*Oreochromis niloticus*)	[35]
		↑	Plasma	4 d	1.96 mg/L	Nile tilapia (*Oreochromis niloticus*)	[26]
	Chlorpyrifos	↓ ↓	Spleen	1, 3, 5, 7 d 3, 5 d	15 *µ*g/L 75 *µ*g/L	Common carp (*Cyprinus carpio*)	[10]
		↑	Kidney	1, 3, 5 d	15, 75 *µ*g/L		
		↓	Plasma	1 d	75 *µ*g/L		
		↓	Plasma	4 d	0.051 mg/mL	Nile tilapia (*Oreochromis niloticus*)	[29]

Contents of complement C3	Chlorpyrifos	—	Spleen	1, 3,5, 7 d	15, 75 *µ*g/L	Common carp (*Cyprinus carpio*)	[10]
		↑	Plasma	1 d	75 *µ*g/L		
		↑ ↓	Kidney	1 d 7 d	15 *µ*g/L 75 *µ*g/L		

Complement C3 expression at mRNA level	Chlorpyrifos	↑	Spleen	1 d	75 *µ*g/L	Common carp (*Cyprinus carpio*)	[10]
		↓		7 d	15, 75 *µ*g/L		
		↑ ↓	Kidney	3 d 5 d	15 *µ*g/L		
		↓		7 d	15, 75 *µ*g/L		

IL-1*β* relative mRNA level		↑	Spleen		1.16, 11.6, 116 *µ*g/L	Common carp (*Cyprinus carpio*)	[36]
		↓	Kidney				
IL-1R relative mRNA level		↑	Spleen		11.6, 116 *µ*g/L		
	Chlorpyrifos	↓ ↑	Kidney	40 d	1.16 *µ*g/L 11.6 *µ*g/L		
IFN-*γ* relative mRNA level		↑	Spleen		11.6 *µ*g/L		
		↓ ↑	Kidney		1.16, 11.6 *µ*g/L 116 *µ*g/L		

Cellular immune response							

Cell proliferation	Diazinon	↓	Lymphocytes	4 d	7.83, 3.91, 1.95 mg/L	Nile tilapia (*Oreochromis niloticus*)	[35]
	Chlorpyrifos	→	Lymphocytes	4 d	0.051, 0.102, 0.255 mg/L	Nile tilapia (*Oreochromis niloticus*)	[29]

WBC Lym Mon Neu Eos Bas	Diazinon	↓ ↓ ↓ ↑ ↑ ↓	Blood	10, 20, 30 d	60, 120 *µ*g/L 120 *µ*g/L	Common carp, (*Cyprinus carpio*)	[37]

WBC	Malathion	↓	Blood	1, 4, 28, 42 d	0.023, 0.46 mg/L	Nile tilapia (*Oreochromis niloticus*)	[1]
	Chlorpyrifos	↑	Blood	14 d	0.040, 0.080 mg/L	Common carp (*Cyprinus carpio*)	[38]

WBC Lym Mon Neu	Diazinon	↓ ↓ ↓ ↑	Blood	7, 15, 30 d	0.1, 0.2 mg/mL	Rainbow trout (*Oncorhynchus mykiss*)	[28]

WBC Lym Mon Neu	Phosalone	↓ ↓ ↑ ↑	Blood	7, 14 d 7, 14 d 14 d 7, 14 d	0.15, 0.30, 0.60 mg/L 0.15, 0.30, 0.60 mg/L 0.30, 0.60 mg/L 0.15, 0.30, 0.60 mg/L	Common carp (*Cyprinus carpio*)	[30]

WBC Lym Mon Eos Neu	Diazinon	↑ → — — ↑	Blood	7 d	0.5, 1 ppm	Iridescent shark (*Pangasius hypophthalmus*)	[39]

Respiratory burst	Diazinon	↑	Splenocytes	4 d	1.96 mg/L	Nile tilapia (*Oreochromis niloticus*)	[26]

Phagocytic index	Diazinon	↓	Blood	4 d	7.83, 3.91 mg/L	Nile tilapia (*Oreochromis niloticus*)	[26]

Phagocytic function							
Gran	Chlorpyrifos	→	Kidney cells		0.1, 1, 10 mg/L	Rainbow fish (*Melanotaenia fluviatilis*)	[63]
Lyn						Silver perch (*Bidyanus bidyanus*)	
Gran						Golden perch (*Macquaria ambigua*)	
Lyn		↓			10 mg/L	Murray cod (*Maccullochella peelii*)	

↑: increase/activation (induction); ↓: inhibition/decrease; →: no effect; —: not detectable; WBC: white blood cell; Lym: lymphocytes; Mon: monocyte; Gra: granulocytes; Eos: eosinophil; Bas: basophil; Neu: neutrophil; d: days.

## References

[1] Al-Ghanim K. A. (2012). Acute toxicity and effects of sub-lethal malathion exposure on biochemical and haematological parameters of *Oreochromis niloticus*. *Scientific Research and Essays*.

[2] Environmental Protection Agency US (EPA) http://npic.orst.edu/ingred/stats.html.

[3] Vittozzi L., Fabrizi L., di Consiglio E., Testai E. (2001). Mechanistic aspects of organophosphorothionate toxicity in fish and humans. *Environment International*.

[4] WHO (1990). *Public Health Impact of Pesticides Used in Agriculture*.

[5] Terry A. V. (2012). Functional consequences of repeated organophosphate exposure: potential non-cholinergic mechanisms. *Pharmacology and Therapeutics*.

[6] Blakley B., Brousseau P., Fournier M., Voccia I. (1999). Immunotoxicity of pesticides: a review. *Toxicology and Industrial Health*.

[7] Costa L. G. (2006). Current issues in organophosphate toxicology. *Clinica Chimica Acta*.

[8] Jokanovi M. (2001). Biotransformation of organophosphorus compounds. *Toxicology*.

[9] Aydın R., Köprücü K. (2005). Acute toxicity of diazinon on the common carp (*Cyprinus carpio* L.) embryos and larvae. *Pesticide Biochemistry and Physiology*.

[10] Li X., Liu L., Zhang Y., Fang Q., Li Y. (2013). Toxic effects of chlorpyrifos on lysozyme activities, the contents of complement C3 and IgM, and IgM and complement C3 expressions in common carp (*Cyprinus carpio* L.). *Chemosphere*.

[11] le Comber S. C., Smith C. (2004). Polyploidy in fishes: Patterns and processes. *Biological Journal of the Linnean Society*.

[12] Volff J.-N. (2005). Genome evolution and biodiversity in teleost fish. *Heredity*.

[13] Hurley I. A., Mueller R. L., Dunn K. A. (2007). A new time-scale for ray-finned fish evolution. *Proceedings of the Royal Society B: Biological Sciences*.

[14] Litman G. W., Cannon J. P., Dishaw L. J. (2005). Reconstructing immune phylogeny: new perspectives. *Nature Reviews Immunology*.

[15] Magnadóttir B. (2006). Innate immunity of fish (overview). *Fish and Shellfish Immunology*.

[16] Quiniou S. M. A., Boudinot P., Bengtén E. (2013). Comprehensive survey and genomic characterization of Toll-like receptors (TLRs) in channel catfish, *Ictalurus punctatus*: identification of novel fish TLRs. *Immunogenetics*.

[17] Gao L., He C., Liu X. (2012). The innate immune-related genes in catfish. *International Journal of Molecular Sciences*.

[18] Saurabh S., Sahoo P. K. (2008). Lysozyme: an important defence molecule of fish innate immune system. *Aquaculture Research*.

[19] Boshra H., Li J., Sunyer J. O. (2006). Recent advances on the complement system of teleost fish. *Fish and Shellfish Immunology*.

[20] Alvarez-Pellitero P. (2008). Fish immunity and parasite infections: from innate immunity to immunoprophylactic prospects. *Veterinary Immunology and Immunopathology*.

[21] Laing K. J., Hansen J. D. (2011). Fish T cells: recent advances through genomics. *Developmental and Comparative Immunology*.

[22] Zwollo P. (2011). Dissecting teleost B cell differentiation using transcription factors. *Developmental and Comparative Immunology*.

[23] Barr M., Mott K., Zwollo P. (2011). Defining terminally differentiating B cell populations in rainbow trout immune tissues using the transcription factor XbpI. *Fish and Shellfish Immunology*.

[24] Sunyer J. O. (2013). Fishing for mammalian paradigms in the teleost immune system. *Nature Immunology*.

[25] Soltani M., Pourgholam R. (2007). Lysozyme activity of grass carp (*Ctenopharingodon idella*) following exposure to sublethal concentrations of organophosphate, diazinón. *Veterinary Research*.

[26] Girón-Pérez M. I., Velázquez-Fernández J., Díaz-Resendiz K. (2009). Immunologic parameters evaluations in Nile tilapia (*Oreochromis niloticus*) exposed to sublethal concentrations of diazinon. *Fish and Shellfish Immunology*.

[27] Banaee M., Sureda A., Mirvaghefi A. R., Ahmadi K. (2011). Effects of diazinon on biochemical parameters of blood in rainbow trout (*Oncorhynchus mykiss*). *Pesticide Biochemistry and Physiology*.

[28] Ahmadi K., Mirvaghefei A. R., Banaee M., Vosoghei A. R. (2014). Effects of long-term diazinon exposure on some immunological and haematological parameters in rainbow trout *Oncorhynchus mykiss* (Walbaum, 1792). *Toxicology and Environmental Health Sciences*.

[29] Díaz-Resendiz K. J. G., Girón-Pérez M. I. (2014). Effect of chlorpyrifos on the immune response of Nile tilapia (*Oreochromis niloticus*). *Revista Bio ciencias*.

[30] Kaya H., Çelik E. Ş., Yılmaz S., Tulgar A., Akbulut M., Demir N. (2014). Hematological, serum biochemical, and immunological responses in common carp (*Cyprinus carpio*) exposed to phosalone. *Comparative Clinical Pathology*.

[31] Khoshbavar-Rostami H. A., Soltani M., Hassan H. M. D. (2006). Immune response of great sturgeon (*Huso huso*) subjected to long-term exposure to sublethal concentration of the organophosphate, diazinón. *Aquaculture*.

[32] Kodama H., Matsuoka Y., Tanaka Y., Liu Y., Iwasaki T., Watarai S. (2004). Changes of C-reactive protein levels in rainbow trout (*Oncorhynchus mykiss*) sera after exposure to anti-ectoparasitic chemicals used in aquaculture. *Fish and Shellfish Immunology*.

[33] Dominguez M., Takemura A., Tsuchiya M., Nakamura S. (2004). Impact of different environmental factors on the circulating immunoglobulin levels in the Nile tilapia, *Oreochromis niloticus*. *Aquaculture*.

[34] Bag M. R., Makesh M., Rajendran K. V., Mukherjee S. C. (2009). Characterization of IgM of Indian major carps and their cross-reactivity with anti-fish IgM antibodies. *Fish and Shellfish Immunology*.

[35] Girón-Pérez M. I., Santerre A., Gonzalez-Jaime F. (2007). Immunotoxicity and hepatic function evaluation in Nile tilapia (*Oreochromis niloticus*) exposed to diazinon. *Fish and Shellfish Immunology*.

[36] Wang X., Xing H., Li X., Xu S., Wang X. (2011). Effects of atrazine and chlorpyrifos on the mRNA levels of IL-1 and IFN-*γ*2b in immune organs of common carp. *Fish and Shellfish Immunology*.

[37] Banaee M., Mirvagefei A. R., Rafei G. R., Majazi Amiri B. (2008). Effect of sub-lethal diazinon concentrations on blood plasma biochemistry. *International Journal of Environmental Research*.

[38] Ural M. Ş. (2013). Chlorpyrifos-induced changes in oxidant/antioxidant status and haematological parameters of *Cyprinus carpio carpio*: ameliorative effect of lycopene. *Chemosphere*.

[39] Hedayati A., Tarkhani R. (2014). Hematological and gill histopathological changes in iridescent shark, *Pangasius hypophthalmus* (Sauvage, 1878) exposed to sublethal diazinon and deltamethrin concentrations. *Fish Physiology and Biochemistry*.

[40] Dutta H. M., Qadri N., Ojha J. (1997). Effect of diazinon on marcophages of bluegill sunfish, *Lepomis macrochirus*: a cytochemical evaluation. *Bulletin of Environmental Contamination and Toxicology*.

[41] Girón-Pérez M. I., Zaitseva G., Casas-Solis J., Santerre A. (2008). Effects of diazinon and diazoxon on the lymphoproliferation rate of splenocytes from Nile tilapia (*Oreochromis niloticus*): the immunosuppresive effect could involve an increase in acetylcholine levels. *Fish and Shellfish Immunology*.

[42] Blalock J. E. (1994). The syntax of immune-neuroendocrine communication. *Immunology Today*.

[43] Kawashima K., Fujii T. (2000). Extraneuronal cholinergic system in lymphocytes. *Pharmacology & Therapeutics*.

[44] Toledo-Ibarra G. A., Rojas-Mayorquín A. E., Girón-Pérez M. I. (2013). Influence of the cholinergic system on the immune response of teleost fishes: potential model in biomedical research. *Clinical and Developmental Immunology*.

[45] Charoenying T., Suriyo T., Thiantanawat A., Chaiyaroj S. C., Parkpian P., Satayavivad J. (2011). Effects of paraoxon on neuronal and lymphocytic cholinergic systems. *Environmental Toxicology and Pharmacology*.

[46] Li Q. (2007). New mechanism of organophosphorus pesticide-induced immunotoxicity. *Journal of Nippon Medical School*.

[47] Li Q., Nagahara N., Takahashi H., Takeda K., Okumura K., Minami M. (2002). Organophosphorus pesticides markedly inhibit the activities of natural killer, cytotoxic T lymphocyte and lymphokine-activated killer: a proposed inhibiting mechanism via granzyme inhibition. *Toxicology*.

[48] Li Q., Hirata Y., Kawada T., Minami M. (2004). Elevated frequency of sister chromatid exchanges of lymphocytes in sarin-exposed victims of the Tokyo sarin disaster 3 years after the event. *Toxicology*.

[49] Li Q., Nakadai A., Ishizaki M. (2005). Dimethyl 2,2-dichlorovinyl phosphate (DDVP) markedly decreases the expression of perforin, granzyme A and granulysin in human NK-92CI cell line. *Toxicology*.

[50] Li Q., Nakadai A., Matsushima H. (2006). Phytoncides (wood essential oils) induce human natural killer cell activity. *Immunopharmacology and Immunotoxicology*.

[51] Kägi D., Vignaux F., Ledermann B. (1994). Fas and perforin pathways as major mechanisms of T cell-mediated cytotoxicity. *Science*.

[52] Smyth M. J., Trapani J. A. (1995). Granzymes: exogenous proteinases that induce target cell apoptosis. *Immunology Today*.

[53] Hirata Y., Inagaki H., Shimizu T. (2006). Expression of enzymatically active human granzyme 3 in *Escherichia coli* for analysis of its substrate specificity. *Archives of Biochemistry and Biophysics*.

[54] Magnarelli G., Fonovich T. (2013). Protein phosphorylation pathways disruption by pesticides. *Advances in Biological Chemistry*.

[55] Esquivel-Sentíes M. S., Barrera I., Ortega A., Vega L. (2010). Organophosphorous pesticide metabolite (DEDTP) induces changes in the activation status of human lymphocytes by modulating the interleukin 2 receptor signal transduction pathway. *Toxicology and Applied Pharmacology*.

[56] Lima A., Vega L. (2005). Methyl-parathion and organophosphorous pesticide metabolites modify the activation status and interleukin-2 secretion of human peripheral blood mononuclear cells. *Toxicology Letters*.

[57] Sitko J. C., Yeh B., Kim M. (2008). SOCS3 regulates p21 expression and cell cycle arrest in response to DNA damage. *Cellular Signalling*.

[58] Niijima H., Nagao M., Nakajima M. (1999). Sarin-like and soman-like organophosphorous agents activate PLC*γ*, in rat brains. *Toxicology and Applied Pharmacology*.

[59] Saleh A. M., Vijayasarathy C., Masoud L., Kumar L., Shahin A., Kambal A. (2003). Paraoxon induces apoptosis in EL4 cells via activation of mitochondrial pathways. *Toxicology and Applied Pharmacology*.

[60] Das G. P., Shaik A. P., Jamil K. (2006). Estimation of apoptosis and necrosis caused by pesticides in vitro on human lymphocytes using DNA diffusion assay. *Drug and Chemical Toxicology*.

[61] Nakadai A., Li Q., Kawada T. (2006). Chlorpyrifos induces apoptosis in human monocyte cell line U937. *Toxicology*.

[62] Chen X. Y., Shao J. Z., Xiang L. X., Liu X. M. (2006). Involvement of apoptosis in malathion-induced cytotoxicity in a grass carp (*Ctenopharyngodon idellu*s) cell line. *Comparative Biochemistry and Physiology, Part C: Toxicology and Pharmacology*.

[63] Harford A. J., O'Halloran K., Wright P. F. A. (2005). The effects of in vitro pesticide exposures on the phagocytic function of four native Australian freshwater fish. *Aquatic Toxicology*.

